# Trends and inequities in severe maternal morbidity in Massachusetts: A closer look at the last two decades

**DOI:** 10.1371/journal.pone.0279161

**Published:** 2022-12-20

**Authors:** Hafsatou Diop, Eugene R. Declercq, Chia-Ling Liu, Howard J. Cabral, Xiaohui Cui, Ndidiamaka Amutah-Onukagha, Audra Meadows

**Affiliations:** 1 Massachusetts Department of Public Health, Boston, Massachusetts, United States of America; 2 Boston University School of Public Health, Boston, Massachusetts, United States of America; 3 Evalogic Services, Inc, Newton, Boston, Massachusetts, United States of America; 4 Tufts University School of Medicine and the Department of Biostatistics, Harvard T. H. Chan School of Public Health, Boston, Massachusetts, United States of America; 5 Department of Obstetrics, Gynecology, and Reproductive Sciences, University of California, San Diego, California, United States of America; Universita degli Studi dell’Insubria, ITALY

## Abstract

It is estimated that 50,000–60,000 pregnant people in the United States (US) experience severe maternal morbidity (SMM). SMM includes life-threatening conditions, such as acute myocardial infarction, acute renal failure, amniotic fluid embolism, disseminated intravascular coagulation, or sepsis. Prior research has identified both rising rates through 2014 and wide racial disparities in SMM. While reducing maternal death and SMM has been a global goal for the past several decades, limited progress has been made in the US in achieving this goal. Our objectives were to examine SMM trends from 1998–2018 to identify factors contributing to the persistent and rising rates of SMM by race/ethnicity and describe the Black non-Hispanic/White non-Hispanic rate ratio for each SMM condition. We used a population-based data system that links delivery records to their corresponding hospital discharge records to identify SMM rates (excluding transfusion) per 10, 000 deliveries and examined the trends by race/ethnicity. We then conducted stratified analyses separately for Black and White birthing people. While the rates of SMM during the same periods steadily increased for all racial/ethnic groups, Black birthing people experienced the greatest absolute increase compared to any other race/ethnic group going from 69.4 in 1998–2000 to 173.7 per 10,000 deliveries in 2016–2018. In addition, we found that Black birthing people had higher rates for every individual condition compared to White birthing people, with rate ratios ranging from a low of 1.11 for heart failure during surgery to a high of 102.4 for sickle cell anemia. Obesity was not significantly associated with SMM among Black birthing people but was associated with SMM among White birthing people [aRR 1.18 (95% CI: 1.02, 1.36)]. An unbiased understanding of how SMM has affected different race/ethnicity groups is key to improving maternal health and preventing SMM and mortality among Black birthing people. SMM needs to be addressed as both a medical and public health challenge.

## Introduction

Deliveries involving severe maternal morbidity (SMM), which is defined as unexpected outcomes of labor and delivery that result in significant short- or long-term consequences to a person’s health, continue to rise in the United States (US) [1]. It is estimated that 50,000–60,000 pregnant people in the US experience SMM [1,2]. SMM includes life-threatening conditions, such as acute myocardial infarction, acute renal failure, amniotic fluid embolism, disseminated intravascular coagulation, or sepsis. Prior research indicated a 75% increase in severe complications during delivery hospitalizations and a 114% increase in severe morbidity during postpartum hospitalizations between the 1998–1999 and 2008–2009 periods [3]. The Centers for Disease Control and Prevention (CDC) reported close to a 200% increase from 49.5 in 1993 to 144.0 per 10,000 deliveries in 2014; this increase was mostly attributed to blood transfusions, which increased from 24.5 in 1993 to 122.3 in 2014 [1].

We described a similar increase in SMM rates at the time of delivery in the Commonwealth of Massachusetts from 129.4 in 2009 to 214.3 per 10,000 in 2018 [4]. One of the Massachusetts Title V program priorities is to eliminate inequities in SMM. An Act to Reduce Racial Inequities in Maternal Health [5] was signed in 2021 in Massachusetts and established a commission to investigate and study ways to reduce or eliminate racial inequities in maternal mortality and SMM in the Commonwealth using evidence-based, best or promising practices. A large body of literature has documented racial/ethnic inequities in reproductive health [6–10] and racism as a fundamental cause of such inequities [11]. A systematic review of 37 studies, mostly conducted in the US and with physicians, found statistically significant evidence of racist beliefs, emotions, or practices among healthcare providers in relation to minority groups in 26 of these studies [12].

A population-based California study found Black non-Hispanics having 44% higher rates of SMM than non-Hispanic Whites, even after adjustment for demographic and medical risk factors [11]. A retrospective study in a single hospital in Illinois that reviewed selected SMM cases indicated that compared to non-Hispanic White, non-Hispanic Black birthing people were more likely to experience a disproportionately high burden of SMM due to preeclampsia and eclampsia (31% vs 18.1%) and were more likely to need improvement in care compared with non-Hispanic White birthing people (53% vs 39.0%) [13]. Black birthing people have not historically received optimal care and racism as a contributor to racial inequities in healthcare is now well recognized [9,14–17].

Globally, where SMM reviews were conducted, the greatest preventable factors identified were provider-related, specifically the lack of identification of “high risk” status, and delays in both diagnosis and treatment [18,19]. In a study that examined preventability of maternal death, near miss, and severe morbidity, provider-related factors were mentioned for approximately 90% of the preventability in all three groups, while system factors were cited in 33% to 47% of preventable events, and patient factors were only reported in 13% to 20% of preventable cases. The major preventability factors at any point in time in the morbidity and mortality continuum were inappropriate and incomplete management, including failure to diagnose, delays in diagnosis, poor communication, and inappropriate referrals [19].

While reducing maternal death and SMM was a Healthy People (HP) 2010 and 2020 goal and remains one of the goals for HP 2030, limited progress has been made in achieving these goals [20], with the release of the 2020 maternal mortality rates reporting the highest US rate since 1968 [21]. To identify factors contributing to the persistent and rising rates of SMM and racial/ethnic inequities, we used a longitudinally linked population-based data system in Massachusetts to examine SMM trends over 20 years, the Black non-Hispanic/White non-Hispanic rate ratio for each SMM condition, and factors associated with SMM by race/ethnicity.

## Materials and methods

### Data source

This study used the Pregnancy to Early Life Longitudinal (PELL) database. The Massachusetts Department of Public Health (MDPH) and the Center for Health information and Analysis (CHIA) are the custodians of the PELL data, which are housed at MDPH. PELL is a population-based data system that links data from live birth certificates and fetal death records (at 350+ grams and/or 20+ weeks gestation) to their corresponding hospital discharge records for the index delivery event, as well as prior and subsequent deliveries. PELL is also linked to non-birth related hospital utilization (hospital admissions, observational stays, and emergency room visits) for birthing individuals and their children over time. Data have been linked for 98% of live births and fetal deaths in Massachusetts for birthing individuals and their children since 1998. These records are linked using LinkPro v3.0 (InfoSoft, Inc., Winnipeg, Manitoba, Canada), a SAS-based deterministic and probabilistic matching program. The core linkage variables include facility code, medical record number, date of birth or delivery, sex, zip code, and birth weight. The longitudinal linkage is based on the parent’s unique encrypted Social Security number (SSN) when available; for records with missing SSN, we used a unique combination of hospital number and medical record number [4]. The longitudinal feature of PELL allows us to examine the hospital contacts that birthing individuals experience at any time during the study period. Approval for the study was granted by the MDPH Institutional Review Board.

### Study sample

**Fig 1:**
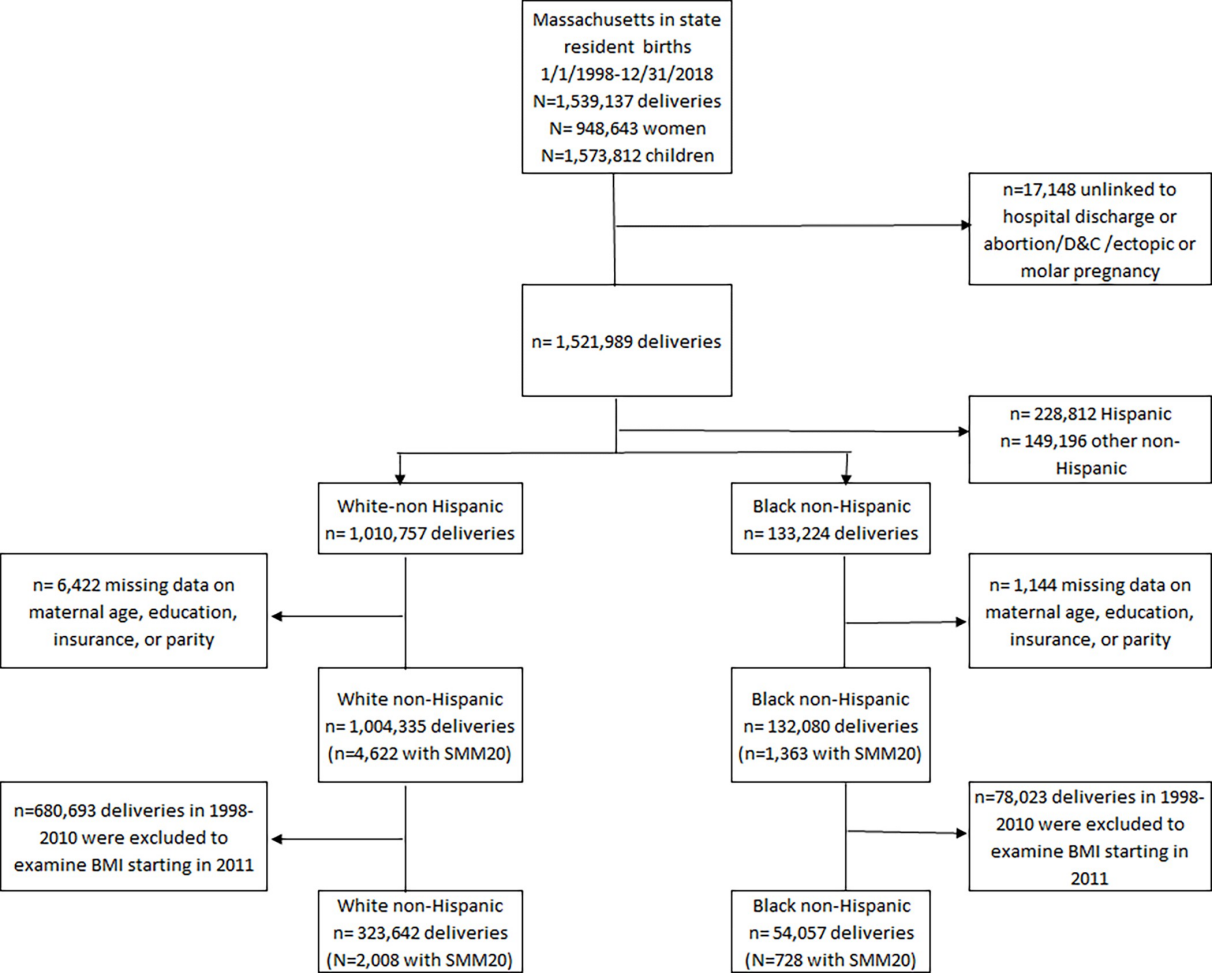
Flow diagram of study sample with inclusion and exclusion criteria.

This analysis included in-state deliveries from January 1, 1998 to December 31, 2018 (N = 1,539,137) to 948,643 Massachusetts residents resulting in 1,573,812 infants. After we excluded delivery records that did not link to a hospital record, early losses, and ectopic and molar pregnancies (n = 17,148), there were 1,521,989 deliveries in our study sample, representing 1,010,757 deliveries to White non-Hispanic, 228,812 to Hispanic, 133,224 to Black non-Hispanic, and 149,196 to all other groups. When examining the factors associated with SMM, we focused our analyses on White non-Hispanic (White) and Black non-Hispanic (Black) birthing people. Deliveries with missing data on maternal age, education, insurance, or parity were excluded. To assess the effects of body mass index (BMI) on SMM, we further restricted our study sample to deliveries in 2011–2018 when the BMI data were available (Fig 1).

### Measures

Our outcome measure was SMM during the delivery hospitalization, based on the algorithm developed as part of an interagency collaboration between the Health Resources and Services Administration (HRSA), CDC, the Agency for Healthcare Research and Quality (AHRQ), and the Alliance for Innovation on Maternal Health (AIM) (Version 07-01-2021). The definition and algorithm rely on 21 conditions or procedures identified through ICD-9 and ICD-10 and procedure codes. Massachusetts hospitals adopted ICD-10 starting in October 2015. The ICD 9 and 10 codes used to identify SMM are included in a supplemental table. We used the CDC algorithm to enhance comparability over time. It includes 21 conditions or procedures including transfusion (SMM21). Because transfusions make up such a large proportion of all SMM cases and the validity of the coding of transfusion has been questioned [22,23], we restricted our analysis to SMM20 to focus on specific SMM conditions, thus excluding transfusion.

Our covariates were chosen based on their relevance in prior research on SMM and included facility annual number of deliveries (<1000, 1000–2000, 2000–4000, and >4000), age (<18, 18–24, 25–29, 30–34, 35–39 and ≥ 40), education (high school or less than high school, some college, college and above), health insurance (private, public, self-pay), year of delivery (1998–2000, 2001–2003, 2004–2006, 2007–2009, 2010–2012, 2013–2015, and 2016–2018), parity (1, 2, and >2), plurality (singletons and multiples), history of hypertension (yes/no), history of diabetes (yes/no), gestational diabetes (yes/no), prenatal care (inadequate, intermediate, adequate and adequate plus) as defined by the Adequacy of Prenatal Care Utilization Index [24]; and, for the 2011–2018 subset, BMI (underweight (<22.5), normal weight (22.5-<25), overweight (25-<30), obese (≥30), missing).

### Statistical analysis

We calculated annual rates of SMM20 (SMM excluding transfusion) per 10,000 deliveries using 3-year rolling averages to smooth individual annual variations and examine the trends by race/ethnicity. Upon examination of the trends, we restricted the remainder of the analyses to the two groups with the highest and lowest rates of SMM overtime, Black and White birthing people. We then examined SMM trends for White and Black birthing people using 3-year rolling averages and Joinpoint regression to assess statistical significance and the annual percentage change for each race/ethnicity group unadjusted for covariates [25]. We examined SMM rates by condition for Black and White birthing people and calculated the Black/White rate ratio for each SMM condition.

Using generalized estimating equations (GEE) Poisson distribution, log link, exchangeable correlation structure models to account for clustering resulting from the inclusion of data from multiple deliveries per birthing person, we examined factors associated with SMM20 for Black and White birthing people, and calculated crude and adjusted risk ratios (aRR) with 95% confidence intervals (CI) sequentially adjusting for covariates. We started our analyses by including race as the main exposure variable. We examined the interaction of race with all covariates and found it not to be significant for education, insurance, year of delivery, parity, history of diabetes, gestational diabetes and adequacy of prenatal care. While prior research has included method of delivery as a covariate [11], we decided not to include it in our model as we were concerned that delivery mode was in the causal pathway and that SMM itself could lead to a cesarean delivery. To further examine the differences observed in these models by race/ethnicity, we then conducted stratified analyses (crude and adjusted with 95% CI) separately for Black and White birthing people. We replicated this stratified analysis to include BMI in the model for the years during which it was available (2011–2018). All analyses were conducted using SAS/STATA14.3.

## Results

**Fig 2:**
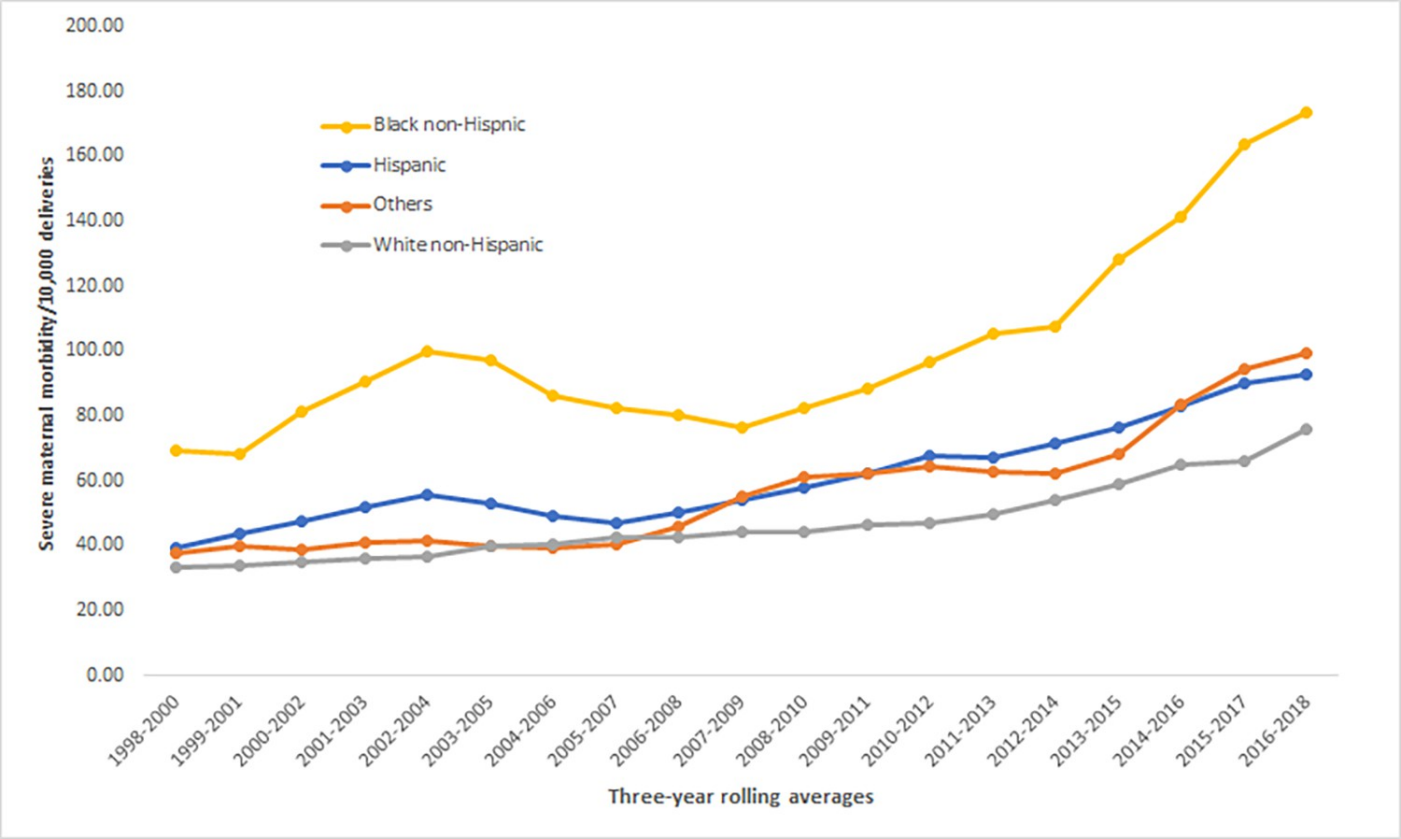
Severe maternal morbidity^a^ by race/ethnicity using three-year rolling average: Massachusetts, 1998–2018. ^a^Severe maternal mortality without blood transfusion (SMM20).

This study included 1,521,989 deliveries to Massachusetts residents during 1998–2018. A substantial number of these deliveries (66.4%) were to White birthing individuals, followed by Hispanic (15.0%), Black (8.8%), and Other non-Hispanic (9.8%). Between the 1998–2000 and 2016–2018 periods, overall SMM20 rates increased significantly from 36.9 to 92.3 per 10,000 deliveries. While the rates of SMM20 during the same periods steadily increased for all racial/ethnic groups, Black birthing people experienced the greatest absolute increase going from 69.4 in 1998–2000 to 173.7 per 10,000 deliveries in 2016–2018. The rates of SMM20 per 10,000 deliveries increased from 33.4 to 76.1 for White, 39.2 to 92.8 for Hispanic, and 37.9 to 99.6 for Other non-Hispanic birthing people between 1998–2000 and 2016–2018 (Fig 2).

**Fig 3:**
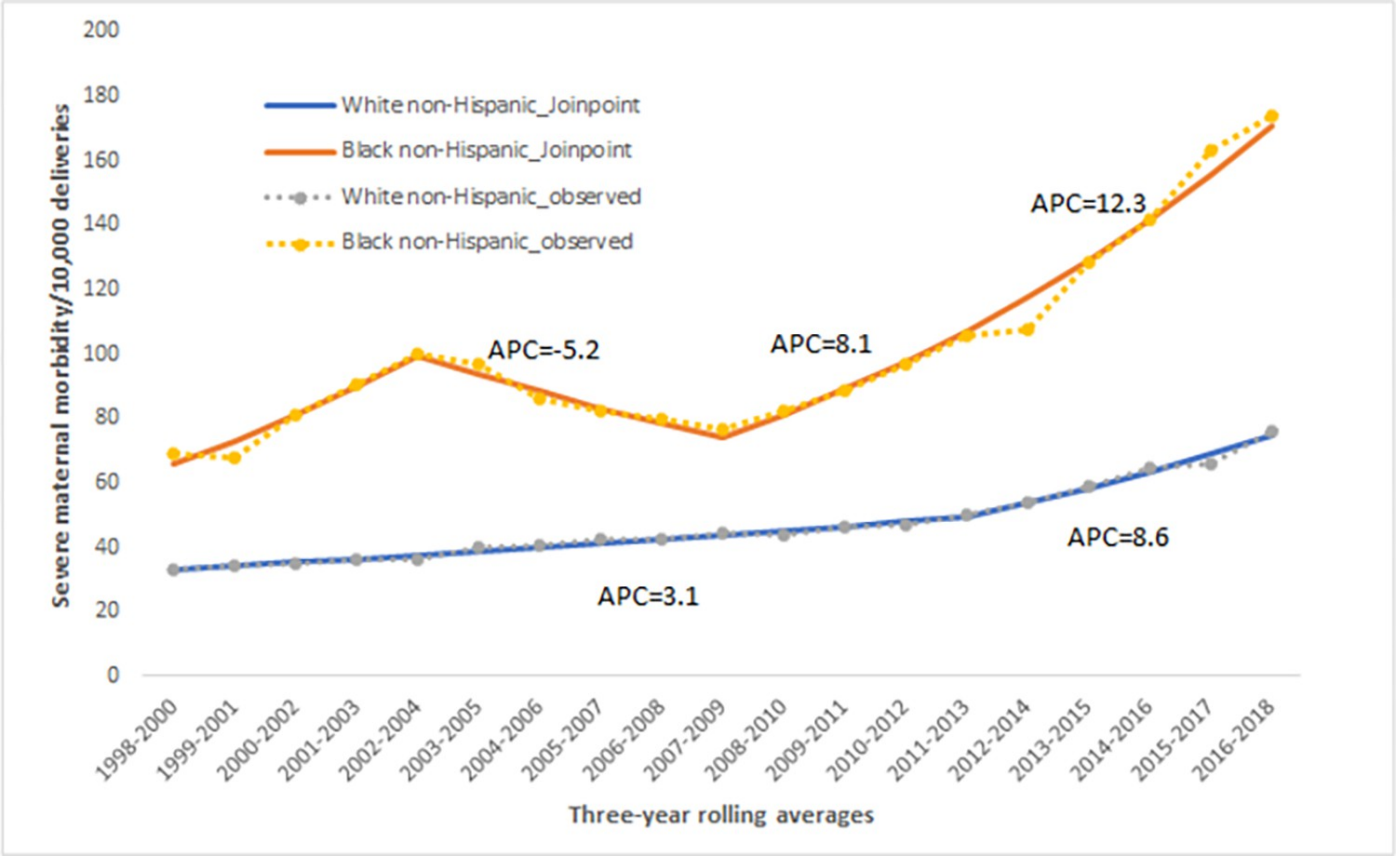
Severe maternal morbidity^a^ for Black and White birthing people: Massachusetts, 1998–2018. ^a^Severe maternal mortality without blood transfusion (SMM20).

Most notably, the rate for Black birthing people in 2016–2018 (173.7 for every 10,000 deliveries) was 128.3% higher than the rate for White, 87.2% and 74.4% higher than the rates for Hispanic and Other non-Hispanic, respectively. The gap between Black and White birthing people in the earlier period (1998–2000) was two-fold. By 2016–2018, the SMM rates for Black birthing people were 2.3 times higher than that of White. Hence, we chose to focus our analysis on better understanding the factors that contribute to SMM in the two racial/ethnic groups with the highest and lowest SMM20 rates. Fig 3 presents the Joinpoint analysis illustrating that SMM20 rates for Black birthing people increased significantly between 1998–2000 and 2002–2004 with an annual percentage change (APC) of 10.6%, declined between 2003–2005 and 2007–2009 with an APC of 5.2%, then steadily increased with an APC of 8.1% and 12.3% between 2008–2010 and 2016–2018. For White birthing people, the rates increased steadily from a lower base from 1998–2000 to 2011–2013 with an APC of 3.1%, and a more pronounced increase between 2012–2014 and 2016–2018 with an APC of 8.6%.

In examining SMM rates for each condition by race/ethnicity, we found that Black birthing people had higher rates for every individual condition (including transfusion in this comparison) compared to White birthing people. For temporary tracheostomy, sepsis, acute renal failure, and pulmonary edema, and sickle cell anemia, the Black/White rate ratios ranged from 3.30 to 102.4. For transfusion, eclampsia, adult respiratory distress syndrome, ventilation, shock, conversion of cardiac rhythm, air and thrombotic embolism, cardiac arrest, and amniotic fluid embolism, the Black/White rate ratios ranged from 2.00 to 2.97 (Table 1).

**Table 1:** Severe maternal morbidity^a^ indicators for White non-Hispanic and Black non-Hispanic birthing people: Massachusetts January 1, 1998-December 31, 2018.

SMM Indicators	Total		White non-Hispanic		Black non-Hispanic		Black/White ratio
	N	Rate per 10,000 deliveries	n	Rate per 10,000 deliveries	n	Rate per 10,000 deliveries	
SMM20^a^	8,428	55.37	4,676	46.26	1399	105.01	2.27
Acute myocardial infarction	37	0.24	22	0.22	7	0.53	2.41
Aneurysm	35	0.23	24	0.24	4	0.30	1.26
Acute renal failure	1,270	8.34	600	5.94	277	20.79	3.50
Adult respiratory distress syndrome	632	4.15	312	3.09	122	9.16	2.97
Amniotic fluid embolism	76	0.50	37	0.37	12	0.90	2.46
Cardiac arrest	91	0.60	41	0.41	13	0.98	2.41
Conversion of cardiac rhythm	77	0.51	33	0.33	11	0.83	2.53
Disseminated intravascular coagulation	2,831	18.60	1,694	16.76	376	28.22	1.68
Eclampsia	799	5.25	439	4.34	123	9.23	2.13
Heart failure during surgery	133	0.87	96	0.95	14	1.05	1.11
Puerperal cerebro-vascular disorders	570	3.75	334	3.30	81	6.08	1.84
Pulmonary edema	739	4.86	365	3.61	159	11.93	3.30
Severe anesthesia complications	337	2.21	200	1.98	48	3.60	1.82
Sepsis	633	4.16	263	2.60	131	9.83	3.78
Shock	508	3.34	259	2.56	87	6.53	2.55
Sickle cell anemia	140	0.92	8	0.08	108	8.11	102.42
Air and thrombotic embolism	333	2.19	202	2.00	59	4.43	2.22
Transfusion	11,812	77.61	6,483	64.14	1706	128.06	2.00
Hysterectomy	836	5.49	461	4.56	120	9.01	1.97
Temporary tracheostomy	17	0.11	8	0.08	4	0.30	3.79
Ventilation	625	4.11	320	3.17	112	8.41	2.66

^a^ Severe maternal morbidity without transfusion (SMM20) counts based on hospital delivery records.

Our stratified analyses using GEE for Black and White are shown in Table 2. Compared to birthing people aged 25–29 (reference group), there are no significant differences in SMM rates among birthing people aged less than 18 years among Whites [aRR 1.01 (95% CI: 0.75, 1.37)] and Blacks [aRR 0.91 (95% CI: 0.62, 1.32)]. However, among birthing people ages 18–24, SMM rates were significantly lower compared the reference group among both Whites [aRR 0.75 (95% CI: 0.67, 0.85)] and Blacks [aRR 0.77 (95% CI: 0.65, 0.92)]. Among White birthing people aged 30 years and older, SMM rates significantly increased with increasing age across all age categories. While among Black birthing people SMM rates show a similar dose response with age, the rates were not significant for the 30–34 age group [aRR 1.10 (95% CI: 0.95–1.28)].

**Table 2:** Characteristics associated with severe maternal morbidity^a^ for White non-Hispanic and Black non-Hispanic birthing people: Massachusetts, January 1, 1998-December 31, 2018.

	White non-Hispanic								Black non-Hispanic							
	No SMM20		Yes SMM20		Crude^b^		Adjusted^b^		No SMM20		Yes SMM20		Crude^b^		Adjusted^b^	
	n	%	n	%	RR	95% CI	aRR	95% CI	n	%	n	%	RR	95% CI	aRR	95% CI
Total	999,713	100.0	4,622	100.0					130,717	100.0	1,363	100.0				
**Facility factors**																
**Average number of deliveries**																
< 1000	189,628	19.0	653	14.1	0.55	0.50–0.60	0.60	0.55–0.66	7,314	5.6	43	3.2	0.53	0.39–0.72	0.54	0.40–0.73
1000- < 2000	255,798	25.6	1,020	22.1	0.64	0.59–0.69	0.67	0.62–0.73	37,136	28.4	302	22.2	0.73	0.63–0.84	0.68	0.59–0.79
2000- < 4000	290,296	29.0	1,294	28.0	0.71	0.66–0.76	0.72	0.67–0.77	36,250	27.7	458	33.6	1.12	0.99–1.27	1.01	0.89–1.15
> 4000	263,991	26.4	1,655	35.8	ref		ref		50,017	38.3	560	41.1	ref		ref	
**Maternal factors**																
**Age**																
< 18	8,994	0.9	45	1.0	1.24	0.92–1.67	1.01	0.75–1.37	3,409	2.6	31	2.3	0.96	0.67–1.39	0.91	0.62–1.32
18–24	140,968	14.1	482	10.4	0.85	0.76–0.95	0.75	0.67–0.85	33,635	25.7	252	18.5	0.79	0.68–0.93	0.77	0.65–0.92
25–29	233,182	23.3	935	20.2	ref		ref		33,924	26.0	321	23.6	ref		ref	
30–34	364,694	36.5	1,602	34.7	1.10	1.01–1.19	1.15	1.06–1.25	33,464	25.6	356	26.1	1.13	0.97–1.31	1.10	0.95–1.28
35–39	206,660	20.7	1,135	24.6	1.37	1.26–1.49	1.44	1.31–1.58	20,178	15.4	295	21.6	1.55	1.32–1.81	1.42	1.21–1.67
≥ 40	45,215	4.5	423	9.2	2.32	2.07–2.60	2.21	1.96–2.49	6,107	4.7	108	7.9	1.87	1.51–2.31	1.55	1.24–1.94
**Education**																
HS or < HS	340,041	34.0	1,474	31.9	0.91	0.85–0.97	1.23	1.13–1.34	75,039	57.4	733	53.8	0.87	0.76–0.99	1.09	0.94–1.27
Some college	146,371	14.6	700	15.1	1.00	0.92–1.09	1.04	0.95–1.13	29,056	22.2	330	24.2	1.01	0.87–1.18	0.98	0.84–1.16
College and above	513,301	51.3	2,448	53.0	ref		ref		26,622	20.4	300	22.0	ref		ref	
**Insurance type at delivery**																
Private	710,440	71.1	3,156	68.3	ref		ref		36,605	28.0	335	24.6	ref		ref	
Public	273,657	27.4	1,349	29.2	1.11	1.04–1.18	1.23	1.13–1.33	91,163	69.7	988	72.5	1.18	1.04–1.33	1.25	1.09–1.44
Self-pay	15,616	1.6	117	2.5	1.67	1.39–2.02	1.10	0.91–1.33	2,949	2.3	40	2.9	1.46	1.06–2.03	1.12	0.81–1.56
**Year of delivery**																
1998–2000	171,403	17.1	568	12.3	ref		ref		16,559	12.7	113	8.3	ref		ref	
2001–2003	164,928	16.5	594	12.9	1.09	0.97–1.22	1.03	0.92–1.15	17,045	13.0	154	11.3	1.32	1.03–1.68	1.25	0.98–1.59
2004–2006	152,248	15.2	613	13.3	1.22	1.08–1.36	1.11	0.99–1.25	17,887	13.7	155	11.4	1.27	1.00–1.61	1.17	0.92–1.49
2007–2009	144,334	14.4	637	13.8	1.34	1.19–1.50	1.22	1.08–1.36	19,378	14.8	147	10.8	1.12	0.88–1.42	1.04	0.81–1.33
2010–2012	129,531	13.0	612	13.2	1.43	1.28–1.60	1.25	1.11–1.40	19,792	15.1	191	14.0	1.43	1.13–1.80	1.27	1.00–1.60
2013–2015	122,174	12.2	717	15.5	1.78	1.59–1.98	1.56	1.39–1.75	19,831	15.2	252	18.5	1.86	1.49–2.33	1.60	1.27–2.02
2016–2018	115,095	11.5	881	19.1	2.30	2.07–2.56	2.03	1.82–2.27	20,225	15.5	351	25.8	2.53	2.05–3.12	2.11	1.70–2.64
**Parity**																
1	457,543	45.8	2,379	51.5	ref		ref		53,317	40.8	594	43.6	ref		ref	
2	352,285	35.2	1,319	28.5	0.73	0.68–0.77	0.62	0.58–0.66	39,623	30.3	319	23.4	0.73	0.64–0.84	0.59	0.52–0.68
>2	189,885	19.0	924	20.0	0.94	0.88–1.02	0.71	0.65–0.77	37,777	28.9	450	33.0	1.08	0.96–1.22	0.72	0.63–0.83
**Plurality**																
Singleton	975,471	97.6	4,205	91.0	ref		ref		128,063	98.0	1,289	94.6	ref		ref	
Multiples	24,242	2.4	417	9.0	3.92	3.55–4.34	3.01	2.71–3.34	2,654	2.0	74	5.4	2.71	2.15–3.41	2.25	1.78–2.85
**History of hypertension** ^c^																
No	941,934	94.2	4,086	88.4	ref		ref		119,380	91.3	1,078	79.1	ref		ref	
Yes	57,779	5.8	536	11.6	2.08	1.89–2.28	1.81	1.65–2.00	11,337	8.7	285	20.9	2.69	2.36–3.07	2.16	1.88–2.49
**History of diabetes** ^d^																
No	973,880	97.4	4,433	95.9	ref		ref		125,645	96.1	1,254	92.0	ref		ref	
Yes	25,833	2.6	189	4.1	1.60	1.39–1.85	1.23	1.05–1.44	5,072	3.9	109	8.0	2.14	1.76–2.60	1.45	1.17–1.81
**Gestational diabetes** ^e^																
No	947,050	94.7	4,289	92.8	ref		ref		121,935	93.3	1,228	90.1	ref		ref	
Yes	52,663	5.3	333	7.2	1.39	1.25–1.56	1.01	0.90–1.14	8,782	6.7	135	9.9	1.51	1.26–1.81	1.00	0.82–1.22
**Adequacy of prenatal Care**																
Inadequate	67,318	6.7	327	7.1	1.52	1.35–1.71	1.96	1.63–2.35	22,380	17.1	242	17.8	1.62	1.37–1.92	1.70	1.29–2.23
Intermediate inadequate	68,221	6.8	206	4.5	0.95	0.82–1.10	1.44	1.28–1.63	9,365	7.2	78	5.7	1.27	1.00–1.62	1.48	1.25–1.76
Adequate	453,406	45.4	1,442	31.2	ref		ref		47,783	36.6	315	23.1	ref		ref	
Adequate Plus	395,911	39.6	2,518	54.5	1.98	1.86–2.11	1.00	0.86–1.15	46,751	35.8	666	48.9	2.13	1.86–2.43	1.24	0.97–1.58
Missing	14,857	1.5	129	2.8	2.70	2.25–3.23	1.75	1.64–1.87	4,438	3.4	62	4.5	2.09	1.60–2.74	1.88	1.64–2.15

^a^Severe maternal mortality without blood transfusion (SMM20).

^b^ RR = Risk ratio; CI = confidence interval; general estimating equation (GEE) was used to adjusted for the effects of multiple deliveries by women; Poisson distribution, log link, exchangeable correlation structure. ref = reference; adjusted for all variables in the table.

^c^Yes = Chronic hypertension reported in the index or prior pregnancies, or pregnancy-related hypertension/preeclampsia/eclampsia reported in prior pregnancies.

^d^Yes = Chronic diabetes reported in the index or prior pregnancies or gestational diabetes reported in prior pregnancies.

^e^Yes = Gestational diabetes reported in the index pregnancy.

While education level was not significantly related to SMM20 for Blacks, high school or less was significantly associated with higher SMM rates among Whites [aRR 1.23 (95% CI: 1.13, 1.34)]. SMM rates for those on public insurance were significantly higher with comparable risks for White [aRR 1.23 (95% CI: 1.13, 1.33)] and Black [aRR 1.25 (95% CI: 1.09, 1.44)] birthing people. Except for 2001–2003 and 2004–2006 periods, SMM rates increased significantly overtime among Whites, while for Blacks, SMM rates increased significantly only between 2010 and 2018. While having multiple births was significantly associated with the risk of SMM for both groups, the risk was more pronounced among Whites [aRR 3.01 (95% CI: 2.71, 3.34)] than Black birthing individuals [aRR 2.25 (95% CI: 1.78, 2.85)]. History of hypertension was significantly associated with higher risk of SMM among Blacks [aRR 2.16 (95% CI: 1.88, 2.49)] and Whites [aRR 1.81 (95% CI: 1.65, 2.00)]. History of diabetes was also associated with higher risk of SMM among Black and White individuals but is more pronounced in Blacks [aRR 1.45 (95% CI: 1.17, 1.81)] compared to Whites [aRR 1.23 (95% CI: 1.05, 1.44)].

Adequacy of prenatal care also showed similar patterns for Blacks and Whites, where inadequate and intermediate inadequate care were significantly associated with higher rates of SMM. The rates of SMM decreased with increasing parity and decreasing facility number of annual deliveries. When we replicated our stratified analyses for White and Black to include BMI in the model for the years for which it was available, we found that BMI was not significantly associated with SMM among Black birthing people, but BMI ≥30 (obese) was significantly associated with SMM [aRR 1.18 (95% CI: 1.02, 1.36)] among White birthing people (Table 3).

**Table 3:** Characteristics associated with severe maternal morbidity^a^ including body mass index for White non-Hispanic and Black non-Hispanic birthing people: Massachusetts February 1, 2011-December 31, 2018.

	White non-Hispanic								Black non-Hispanic							
	No SMM20		Yes SMM20		Crude^b^		Adjusted^b^		No SMM20		Yes SMM20		Crude^b^		Adjusted^b^	
	n	%	n	%	RR	95% CI	aRR	95% CI	n	%	n	%	RR	95% CI	aRR	95% CI
Total	321,634	100.0	2,008	100.0					53,329	100.0	728	100.0				
**Facility factors**																
**Average number of deliveries**																
< 1000	55,970	17.4	253	12.6	0.50	0.43–0.58	0.54	0.47–0.63	3,171	5.9	25	3.4	0.51	0.34–0.76	0.54	0.36–0.81
1000- < 2000	73,829	23.0	357	17.8	0.54	0.47–0.61	0.57	0.50–0.65	16,516	31.0	151	20.7	0.58	0.48–0.71	0.58	0.47–0.71
2000- < 4000	108,106	33.6	640	31.9	0.65	0.59–0.73	0.69	0.62–0.77	15,547	29.2	265	36.4	1.07	0.91–1.27	1.02	0.86–1.22
> 4000	83,729	26.0	758	37.7	ref		ref		18,095	33.9	287	39.4	ref		ref	
**Maternal factors**																
**Age**																
< 18	1,402	0.4	11	0.5	1.52	0.84–2.75	1.17	0.64–2.14	610	1.1	10	1.4	1.36	0.73–2.55	1.25	0.65–2.37
18–24	36,957	11.5	160	8.0	0.84	0.70–1.01	0.75	0.62–0.92	11,245	21.1	111	15.2	0.83	0.65–1.05	0.81	0.63–1.04
25–29	74,745	23.2	386	19.2	ref		ref		14,075	26.4	167	22.9	ref		ref	
30–34	126,016	39.2	743	37.0	1.14	1.01–1.29	1.18	1.04–1.34	15,066	28.3	213	29.3	1.18	0.96–1.44	1.17	0.96–1.44
35–39	67,715	21.1	516	25.7	1.47	1.29–1.68	1.52	1.32–1.75	9,365	17.6	168	23.1	1.50	1.21–1.85	1.44	1.15–1.79
≥ 40	14,799	4.6	192	9.6	2.49	2.09–2.95	2.34	1.96–2.79	2,968	5.6	59	8.1	1.65	1.23–2.22	1.44	1.06–1.96
**Education**																
High school (HS) or < HS	57,117	17.8	367	18.3	0.99	0.88–1.11	1.14	0.99–1.32	20,401	38.3	270	37.1	0.88	0.73–1.06	1.01	0.82–1.24
Some college	77,025	23.9	422	21.0	0.84	0.75–0.94	0.94	0.83–1.07	19,942	37.4	263	36.1	0.88	0.73–1.06	0.98	0.80–1.19
College and above	187,492	58.3	1,219	60.7	ref		ref		12,986	24.4	195	26.8	ref		ref	
**Insurance type at delivery**																
Private	202,925	63.1	1,222	60.9	ref		ref		11,584	21.7	150	20.6	ref		ref	
Public	107,906	33.5	693	34.5	1.06	0.97–1.17	1.31	1.17–1.47	39,671	74.4	545	74.9	1.06	0.88–1.27	1.25	1.02–1.52
Self-pay	10,803	3.4	93	4.6	1.42	1.15–1.76	1.06	0.86–1.32	2,074	3.9	33	4.5	1.22	0.84–1.78	1.10	0.76–1.60
**Year of delivery**																
2011–2012	84,365	26.2	410	20.4	ref		ref		13,273	24.9	125	17.2	ref		ref	
2013–2014	82,101	25.5	472	23.5	1.18	1.04–1.35	1.22	1.07–1.40	13,283	24.9	152	20.9	1.20	0.96–1.52	1.18	0.94–1.50
2015–2016	79,576	24.7	526	26.2	1.36	1.20–1.55	1.45	1.27–1.65	13,235	24.8	210	28.8	1.67	1.34–2.07	1.64	1.32–2.04
2017–2018	75,592	23.5	600	29.9	1.63	1.44–1.84	1.69	1.48–1.92	13,538	25.4	241	33.1	1.86	1.51–2.30	1.77	1.42–2.20
**Parity**																
1	150,281	46.7	1,051	52.3	ref		ref		21,238	39.8	326	44.8	ref		ref	
2	113,448	35.3	561	27.9	0.71	0.64–0.79	0.61	0.55–0.68	16,270	30.5	173	23.8	0.70	0.59–0.84	0.57	0.47–0.69
>2	57,905	18.0	396	19.7	0.98	0.87–1.10	0.71	0.63–0.81	15,821	29.7	229	31.5	0.96	0.81–1.13	0.64	0.53–0.77
**Plurality**																
Singleton	314,488	97.8	1,852	92.2	ref		ref		52,234	97.9	693	50.8	ref		ref	
Multiples	7,146	2.2	156	7.8	3.63	3.09–4.27	2.73	2.32–3.23	1,095	2.1	35	2.6	2.34	1.67–3.28	2.03	1.44–2.85
**History of hypertension** ^c^																
No	295,927	92.0	1,725	85.9	ref		ref		47,394	88.9	549	75.4	ref		ref	
Yes	25,707	8.0	283	14.1	1.86	1.64–2.11	1.68	1.47–1.93	5,935	11.1	179	24.6	2.53	2.14–3.00	2.20	1.83–2.65
**History of diabetes** ^d^																
No	310,682	96.6	1,910	95.1	ref		ref		50,931	95.5	664	25.5	ref		ref	
Yes	10,952	3.4	98	4.9	1.45	1.18–1.77	1.22	0.98–1.52	2,398	4.5	64	2.5	2.02	1.57–2.60	1.42	1.07–1.88
**Gestational diabetes** ^e^																
No	299,858	93.2	1,841	91.7	ref		ref		49,190	92.2	645	88.6	ref		ref	
Yes	21,776	6.8	167	8.3	1.25	1.06–1.46	0.96	0.81–1.33	4,139	7.8	83	11.4	1.51	1.20–1.90	1.08	0.84–1.38
**Body Mass Index**																
Underweight (<22.5)	107,563	33.4	616	30.7	1.00	0.88–1.14	1.02	0.89–1.15	11,087	20.8	130	17.9	0.91	0.71–1.17	0.97	0.75–1.24
Normal weight (22.5-<25)	67,958	21.1	388	19.3	ref		ref		8,958	16.8	116	15.9	ref		ref	
Overweight (25-<30)	73,769	22.9	455	22.7	1.08	0.94–1.24	1.06	0.92–1.21	14,648	27.5	180	24.7	0.96	0.76–1.21	0.89	0.71–1.12
Obese (≥30)	57,451	17.9	427	21.3	1.30	1.13–1.49	1.18	1.02–1.36	14,246	26.7	239	32.8	1.30	1.04–1.62	1.04	0.83–1.31
Missing	14,893	4.6	122	6.1	1.43	1.16–1.75	1.37	1.11–1.70	4,390	8.2	63	8.7	1.11	0.82–1.51	1.08	0.79–1.48
**Adequacy of prenatal care**																
Inadequate	24,687	7.7	170	8.5	1.61	1.36–1.91	1.62	1.29–2.03	9,899	18.6	146	20.1	1.50	1.21–1.87	1.33	0.93–1.91
Intermediate inadequate	19,741	6.1	100	5.0	1.19	0.97–1.47	1.58	1.33–1.88	3,657	6.9	46	6.3	1.30	0.94–1.78	1.40	1.12–1.75
Adequate	133,406	41.5	566	28.2	ref		ref		17,540	32.9	170	23.4	ref		ref	
Adequate plus	132,595	41.2	1,081	53.8	1.91	1.72–2.11	1.26	1.02–1.56	19,523	36.6	329	45.2	1.71	1.42–2.05	1.25	0.90–1.72
Missing	11,205	3.5	91	4.5	1.90	1.52–2.36	1.71	1.54–1.89	2,710	5.1	37	5.1	1.40	0.99–1.98	1.54	1.28–1.86

^a^Severe maternal mortality without blood transfusion (SMM20).

^b^ RR = Risk ratio; CI = confidence interval; general estimating equation (GEE) was used to adjusted for the effects of multiple deliveries by women; Poisson distribution, log link, exchangeable correlation structure. ref = reference; adjusted for all variables in the table.

^c^Yes = Chronic hypertension reported in the index or prior pregnancies, or pregnancy-related hypertension/preeclampsia/eclampsia reported in prior pregnancies.

^d^Yes = Chronic diabetes reported in the index or prior pregnancies or gestational diabetes reported in prior pregnancies.

^e^Yes = Gestational diabetes reported in the index pregnancy.

## Discussion

Our study shows that while the rates of SMM for all race/ethnic groups have continued to rise over the last two decades, Black birthing people have persistently experienced the highest rates of any race/ethnic group, and those rates increased by 150% between the 1998–2000 and 2016–2018 periods, widening an already large Black/White difference. This finding is consistent with other studies. [26,27] Our study also found that for every SMM condition, Black birthing people had much higher rates of SMM, with gaps between Black and White, ranging from a low of 1.11 for heart failure during surgery to a high of 102.4 for sickle cell anemia. Prior research suggests that while Black birthing people are more likely to develop these conditions, they are less likely to have their conditions adequately managed, and more likely to have complications and mortality from these conditions [28]. In our adjusted model, in additional to being Black, factors significantly associated with SMM included multiple birth, history of hypertension, history of diabetes, being on public insurance, having a high school or less than high school education, and inadequate prenatal care. Facility number of annual deliveries, age, parity, and year of delivery were also associated with increased risk of SMM for Black birthing people, suggesting a dose response.

Our stratified analyses sequentially adjusting for covariates showed that education (high school or less) was significantly associated with SMM among Whites, while the risk of SMM among those who are on public insurance was comparable among White and Black birthing people. The aRR for multiple births was higher among White people, while the aRRs for history of hypertension and diabetes were higher among Black birthing people. When we include BMI in the model, we found that BMI was not significantly associated with SMM among Black birthing people, but BMI ≥30 (obese) was significantly associated with SMM among White birthing people.

Most SMM conditions are interrelated, preventable, and may be related to preexisting conditions or develop secondary to diseases of pregnancy [29]. For example, since eclampsia is complication of severe preeclampsia, understanding and treating preeclampsia can prevent SMM due to eclampsia and mortality [30,31].

Our study showed that the Black/White rate ratio for eclampsia was 2.13, which is consistent with findings from the CDC that reported a 3.1 times increased incidence of preeclampsia/eclampsia in Black patients compared to White patients [28]. These findings are disturbing since treatment for preeclampsia and eclampsia is available even in developing countries. Magnesium sulfate (MgSO4), the drug of choice for treating and preventing eclampsia [32–34], has been recognized by the World Health Organization (WHO) as the most efficient and safest medication for the treatment of preeclampsia and eclampsia; WHO has made it available on the Essential Medicines List for this explicit use. Findings from clinical trials conducted between 1995 and 2002, indicated that MgSO4 was the most effective when compared with other treatments, including diazepam and phenytoin. Patients treated with MgSO4 had a 52% lower recurrence of eclampsia than those treated with diazepam and 67% lower recurrence of convulsion than those treated with phenytoin [32]. MgSO4 was also found to decrease the occurrence of eclampsia by more than 50% and maternal deaths by 46% [35]. In addition, MgSO4 is cost-effective and approximately costs $0.10/ml [33].

Our study also found that Black birthing individuals experienced a higher prevalence of acute renal failure (20.8/10,000 deliveries) compared to Whites (5.9/10,000 deliveries). While acute renal failure is a rare event, it can be associated with substantial morbidity and mortality. The etiology of renal failure is complex and could include prerenal factors, which lead to decreased renal perfusion, intrarenal factors which affect the renal parenchyma, or postrenal usually due to obstructions. Prerenal causes of acute renal failure could result from hypovolemia due to obstetric hemorrhage (abortion, placenta previa, placental abruption, uterine rupture or postpartum hemorrhage), but may also arise from sepsis and severe cases of hyperemesis gravidarum, leading to ischemia from decreased renal perfusion and hypotension, and from amniotic fluid embolism leading to disseminated intravascular coagulation (DIC), cardiac dysfunction, and hemorrhage causing intravascular volume depletion and reduced renal perfusion. About 40% of acute renal failure in pregnancy is caused by severe preeclampsia and hemolysis, elevated liver enzymes, and low platelet count (HELLP) syndrome, which can be prevented with adequate and timely management of the pregnant patient [36].

Our study shows that compared to Whites, for every 10,000 deliveries, Black birthing people were more likely to have a diagnosis of DIC (28.2 vs. 16.8), pulmonary edema (11.9 vs. 3.6), and sepsis (9.8 vs. 2.6). While sepsis is a common cause of DIC, other leading causes of DIC include preeclampsia, HELLP syndrome, placental abruption and postpartum hemorrhage [37]. An observational survey conducted in Japan showed that among 1,895 patients with sepsis who were treated in intensive care units, 29% were diagnosed with sepsis-induced DIC [37]. Hence, adequate and timely management of conditions such as sepsis, preeclampsia, HELLP syndrome, and postpartum hemorrhage is key to preventing DIC. Other prevention efforts for SMM should also be placed in the context of public health interventions.

A study indicated that late-season influenza infection between April and June, is associated with a higher risk of SMM and sepsis in pregnant patients and recommends providers remain vigilant as vaccination, early identification and treatment of influenza is associated with improved outcomes among pregnant people [38]. Other studies of pregnant patients with influenza documented increased risk of SMM, increased incidence of hospitalization [39], ICU admission, pneumonia, ventilator support, and maternal or fetal death [40,41], which could all be prevented via screenings and vaccination. A Massachusetts study indicated vaccination rates were significantly higher among pregnant patients whose provider offered or recommended the seasonal (75.8%) and pH1N1 (68.1%) vaccines compared with those who did not receive a recommendation (32.4% and 8.6%, respectively) [41].

Prior research has documented concerning patterns of delay in recognition of hemorrhage, hypertensive crisis, sepsis, venous thromboembolism, and heart failure [42]. Utilization of evidence-based protocols, triggers, bundles, and checklists can improve timely diagnosis and treatment and prevent or minimize the severity of illness, but also enable interdisciplinary, patient-centered care [43]. To facilitate timely recognition, diagnosis and treatment for patients who advance to critical illness, early-warning systems have been proposed by a multidisciplinary working group convened by the National Partnership for Maternal Safety. The Maternal Early Warning Criteria includes a list of abnormal parameters that indicate the need for urgent evaluation by a clinician with the ability to escalate care as needed [42].

It is also documented that even at comparable levels of access to care, people of color experienced a lower quality of health services and are less likely to receive even routine medical procedures than White Americans [44]. This is reflected in our findings showing that Blacks had higher rates for every SMM condition, which suggests a lack of timely diagnosis, failure to treat, poorer quality medical care due to racism or a combination of these. Black people have been found to be substantially more vulnerable to receiving poor quality of care [44]. Provider held unconscious bias was identified by the National Academy of Medicine as one of the three root causes of racial ethnic inequities; the other two being limited access to care and lack of trust in the healthcare system [9]. Providers’ bias in clinical encounters, has led to people of color receiving inferior medical care and fewer procedures compared to Whites across nearly every type of diagnostic and treatment interventions [31]. Black, Hispanic, and Native birthing people also report higher rates of mistreatment in perinatal care settings [45–47].

Our study has several limitations that should be noted. We relied on linked vital statistics and administrative data and these may not always capture the nuances of severe morbidities, though the algorithm used for measuring SMM has been subject to considerable analysis and testing [3]. Also, our study may not be generalizable to other states or the US because the Massachusetts birthing population is not representative of the nation, with a smaller proportion of births to Blacks (10.1% vs 15.0% in U.S.) than the national average. Our sample also has a larger proportion of births to older birthing individuals (57% age 30+) than the U.S. (42% 30+) [4]. Our study also may not be comparable to other SMM analyses since it does not include transfusion, though we think the greater reliability of measurement associated with the SMM20 justifies that limitation [22]. Finally, we included only delivery hospitalizations and did not capture birthing people hospitalized prenatally or postpartum to assess for SMM conditions that were not also present at delivery. We also included limited facility characteristics in this analysis although key institutional differences in where Black and White birthing people deliver have been previously reported [48]. A recent published study found that by using a population-based, longitudinally linked dataset to examine prenatal and postpartum hospitalizations, an additional 22% of cases of SMM between 2009 and 2018 were identified [4].

Our study has several strengths. First, it relies on a large population-based dataset that longitudinally linked birth and fetal death certificates with hospital discharge data, which allows for better identification of deliveries, since hospital discharge data do not have date of delivery and rely on ICD and procedure codes to identify deliveries. Our study relied on a robust linkage; data have been linked for 98% of live births and fetal deaths in Massachusetts for birthing individuals and their children since 1998. Our study was also based on two decades worth of data and allowed us to identify persistent trends. We conducted both adjusted and stratified analyses.

The fact that the rates for White non-Hispanic and all other race/ethnic groups were largely similar during our study period and continued to show similar patterns over time, while the rates and the gaps have continued to worsen for Black birthing people, indicates that Black birthing people have apparently not benefited from improved medical knowledge and care. It has been long recognized that race itself is not a risk factor; exposure to racism is the risk factor as it leads to discriminatory beliefs and behaviors toward Black birthing people [49]. In their review, Diop et al demonstrated the importance of addressing experiences of bias and racism directly with patients and the unique role healthcare providers can play. They proposed a framework of trauma-informed care, structural competency, provider bias and intersectionality when discussing patients’ experiences of racism [50]. Hardeman et al, have proposed to couple critical race theory with the previously described relationship centered care to improve the clinical experiences of Black birthing people [51,52].

An unbiased understanding of how SMM has affected different race ethnicity groups is key to improving maternal health and preventing SMM and mortality among Black pregnant people. SMM needs to be addressed as both a medical and public health challenge. This comprises identification of not only clinical but also social drivers of maternal morbidity, including racism, and other modifiable factors [31]. It is now recognized that an inpatient- or delivery- focused response alone is insufficient to adequately recognize, prevent or respond to significant morbidity during the antepartum period. To eliminate inequities, Carmichael et al. conceptualized SMM within both multidimensional structural and societal factors and the described pathways from specific clinical precursors to specific SMM indicators that can potentially prevent acute progression of disease to life-threatening conditions [31].

In a prior study, we suggested a need to ensure outpatient implementation and surveillance of care quality activities to identify and prevent morbidity from severe hypertension, sepsis and preeclampsia [4]. It is time for healthcare providers at all levels to acknowledge the historical legacy of racism and build a culture that centers on equity. State perinatal quality collaboratives should work with state health departments, Medicaid agencies, and community members with lived experience to better understand the degree to which implicit bias, racism, and discrimination affect Black birthing people at the community and institutional level and identify potential solutions.

## Supporting information

S1 Table ICD 9 and ICD 10 codes used to identify severe maternal morbidity. (DOCX)
