# Supplementary material for: Trends and inequities in severe maternal morbidity in Massachusetts: A closer look at the last two decades
Source: PLoS One. 2022 Dec 20;17(12):e0279161. doi: 10.1371/journal.pone.0279161 (PMC9767362; doi:10.1371/journal.pone.0279161)
Supplement: S1 Table — (DOCX) [file pone.0279161.s001.docx]

**Table 1 ICD 9 and ICD 10 Codes used for Severe Maternal Morbidity Identification**

| ICD 9 | ICD 10 | ICD 10 Description |
| --- | --- | --- |
| 410.xx | I21.xx | Acute myocardial infarction |
|  | I22.x | Subsequent ST elevation (STEMI) and non-ST elevation (NSTEMI) myocardial infarction |
| 441.xx | I71.xx | Aortic aneurysm and dissection |
|  | I79.0 | Aneurysm of aorta in diseases classified elsewhere |
| 584.5 - 584.9 | N17.x | Acute kidney failure |
| 669.3x | O90.4 | Postpartum acute kidney failure |
| 518.5x | J80 | Acute respiratory distress syndrome |
| 518.81 | J95.1 | Acute pulmonary insufficiency following thoracic surgery |
| 518.82 | J95.2 | Acute pulmonary insufficiency following nonthoracic surgery |
| 518.84 | J95.3 | Chronic pulmonary insufficiency following surgery |
| 799.1 | J95.82x | Postprocedural respiratory failure |
|  | J96.0x | Acute respiratory failure |
|  | J96.2x | Acute and chronic respiratory failure |
|  | J96.9x | Respiratory failure, unspecified |
|  | R06.03 | Acute respiratory distress syndrome |
|  | R09.2 | Respiratory arrest |
| 673.1x | O88.1x | Amniotic fluid embolism |
| 427.41 | I46.x | Cardiac arrest |
| 427.42 | I49.0x | Ventricular fibrillation and flutter |
| 427.5 |  |  |
| 99.6x | 5A2204Z | Restoration of cardiac rhythm, single |
|  | 5A12012 | Performance of cardiac output, single, manual |
| 286.6 | D65 | Disseminated intravascular coagulation [defibrination syndrome] |
| 286.9 | D68.8 | Other specified coagulation effects |
| 641.3x | D68.9 | Coagulation defect, unspecified |
| 666.3X | O45.002, O45.003, O45.009 | Premature separation of placenta with coagulation defect, unspecified; second, third, unspecified trimester |
|  | O45.012, O45.013, O45.019 | Premature separation of placenta with afibrinogenemi; second, third, unspecified trimester |
|  | O45.022, O45.023, O45.029 | Premature separation of placenta with disseminated intravascular coagulation; second, third, unspecified trimester |
|  | O45.092, O45.093, O45.O99 | Premature separation of placenta with other coagulation defect; second, third, unspecified trimester |
|  | O46.002, O46.003, O46.009 | Antepartum hemorrhage with coagulation defect, unspecified; second, third, unspecified trimester |
|  | O46.012, O46.013, O46.019 | Antepartum hemorrhage with afibrinogenemia; second, third, unspecified trimester |
|  | O46.022, O46.023, O46.029 | Antepartum hemorrhage with dissem intravascular coagulation; second, third, unspecified trimester |
|  | O46.092, O46.093, O46.099 | Antepartum hemorrhage with other coagulation defect; second, third, unspecified trimester |
|  | O67.0 | Intrapartum hemorrhage with coagulation defect |
|  | O72.3 | Postpartum coagulation defects |
| 642.6x | O15.x | Eclampsia |
| 997.1 | I97.12x | Postprocedural cardiac arrest |
|  | I97.13x | Postprocedural heart failure |
|  | I97.710 | Intraoperative cardiac arrest during cardiac surgery |
|  | I97.711 | Intraoperative cardiac arrest during other surgery |
| 046.3 | A81.2 | Progressive multifocal leukoencephalopathy |
| 348.39 | G45.x | Transient cerebral ischemic attacks and related syndromes |
| 362.34 | G46.x | Vascular syndromes of brain in cerebrovascular diseases |
| 430.xx | G93.49 | Other encephalopathy |
| 431.xx | H34.0x | Transient retinal artery occlusion |
| 432.xx | I60.xx | Nontraumatic subarachnoid hemorrhage |
| 433.xx | I61.xx | Nontraumatic intracerebral hemorrhage |
| 434.xx | I62.xx | Other and unspecified nontraumatic intracranial hemorrhage |
| 435.xx | I63.xx | Cerebral infarction |
| 436.xx | I65.xx | Occlusion and stenosis of precerebral arteries, not resulting in cerebral infarction |
| 437.xx | I66.xx | Occlusion and stenosis of cerebral arteries, not resulting in cerebral infarction |
| 671.5x | I67.xx | Other cerebrovascular diseases |
| 674.0x | I68.xx | Cerebrovascular disorders in diseases classified elsewhere |
| 997.02 | O22.50 | Cerebral venous thrombosis in pregnancy, unspecified trimester |
|  | O22.52 | Cerebral venous thrombosis in pregnancy, second trimester |
|  | O22.53 | Cerebral venous thrombosis in pregnancy, third trimester |
|  | I97.81x | Intraoperative cerebrovascular infarction |
|  | I97.82x | Postprocedural cerebrovascular infarction |
|  | O87.3 | Cerebral venous thrombosis in the puerperium |
| 518.4 | J81.0 | Acute pulmonary edema |
| 428.0 | I50.1 | Left ventricular failure |
| 428.1 | I50.20 | Unspecified systolic (congestive) heart failure |
| 428.20 | I50.21 | Acute systolic (congestive) heart failure |
| 428.21 | I50.23 | Acute on chronic systolic (congestive) heart failure |
| 428.23 | I50.30 | Unspecified diastolic (congestive) heart failure |
| 428.30 | I50.31 | Acute diastolic (congestive) heart failure |
| 428.31 | I50.33 | Acute on chronic diastolic (congestive) heart failure |
| 428.33 | I50.40 | Unspecified combined systolic (congestive) and diastolic (congestive) heart failure |
| 428.40 | I50.41 | Acute combined systolic (congestive) and diastolic (congestive) heart failure |
| 428.41 | I50.43 | Acute on chronic combined systolic (congestive) and diastolic (congestive) heart failure |
| 428.43 | I50.810 | Right heart failure, unspecified |
|  | I50.811 | Acute right heart failure |
|  | I50.813 | Acute on chronic right heart failure |
|  | I50.814 | Right heart failure due to left heart failure |
|  | I50.82 | Biventricular heart failure |
|  | I50.83 | High output heart failure |
|  | I50.84 | End stage heart failure |
|  | I50.89 | Other heart failure |
| 428.9 | I50.9 | Heart failure, unspecified |
| 668.0x | O29.112, O29.113, O29.119 | Cardiac arrest due to anesthesia during pregnancy; second, third, unspecified trimester |
| 668.1x | O29.122, O29.123, O29.129 | Cardiac failure due to anesthesia during pregnancy; second, third, unspecified trimester |
| 668.2x | O29.192, O29.193, O29.199 | Oth cardiac comp of anesth during preg; second, third, unspecified trimester |
| 995.4 | O29.212, O29.213, O29.219 | Cerebral anoxia due to anesth during preg; second, third, unspecified trimester |
| 995.86 | O29.292, O29.293, O29.299 | Oth cnsl comp of anesth during pregnancy; second, third, unspecified trimester |
|  | O74.0 | Aspiration pneumonitis due to anesthesia during labor and delivery |
|  | O74.1 | Other pulmonary complications of anesthesia during labor and delivery |
|  | O74.2 | Cardiac complications of anesthesia during labor and delivery |
|  | O74.3 | Central nervous system complications of anesthesia during labor and delivery |
|  | O89.0x | Pulmonary complications of anesthesia during the puerperium |
|  | O89.1 | Cardiac complications of anesthesia during the puerperium |
|  | O89.2 | Central nervous system complications of anesthesia during the puerperium |
|  | T88.2XXA | Shock due to anesthesia, initial encounter |
|  | T88.3XXA | Malignant hyperthermia due to anesthesia, initial encounter |
| 038.xx | O85 | Puerperal sepsis |
| 449 | O86.04 | Sepsis following an obstetrical procedure |
| 785.52 |  |  |
| 995.91 |  |  |
| 995.92 | T81.44XA | Sepsis following a procedure |
| 998.02 | R65.20 | Severe sepsis without septic shock |
| 670.2x | R65.21 | Severe sepsis with septic shock |
|  | A40.x | Streptococcal sepsis |
|  | A41.x | Other sepsis |
|  | A32.7 | Listerial sepsis |
| 669.1x | O75.1 | Shock during or following labor and delivery |
|  | R57.x | Shock, not elsewhere classified |
| 785.50 |  |  |
| 785.51 | T78.2XXA | Anaphylactic shock, unspecified, initial encounter |
| 785.59 |  |  |
| 995.0 | T88.6XXA | Anaphylactic reaction due to adverse effect of correct drug or medicament properly administered, initial encounter |
|  | T81.10XA | Postprocedural shock unspecified, initial encounter |
| 998.0x | T81.11XA | Postprocedural cardiogenic shock, initial encounter |
| 998.00 | T81.19XA | Other postprocedural shock, initial encounter |
| 998.01 |  |  |
| 998.09 |  |  |
| 282.42 | D57.0x | Hb-SS disease with crisis |
| 282.62 | D57.21x | Sickle cell/Hb-C disease with crisis |
| 282.64 | D57.41x | Sickle cell thalassemia with crisis |
| 282.69 | D57.81x | Other sickle cell disorders with crisis |
| 289.52 |  |  |
| 415.0 | I26.x | Pulmonary embolism |
| 415.1x | O88.012-O88.03 | Obstetric air embolism |
| 673.0x | O88.212-O88.23 | Obstetric thromboembolism |
| 673.2x | O88.312-O88.33 | Obstetric pyemic and septic embolism |
| 673.3x | O88.812-O88.83 | Other obstetric embolism |
| 673.8x | T80.0XXA | Air embolism following infusion, transfusion and therapeutic injection, initial encounter |
| 99.0x | 30230H0 | Transfusion of autologous whole blood into peripheral vein, open approach |
|  | 30230K0 | Transfusion of autologous frozen plasma into peripheral vein, open approach |
|  | 30230L0 | Transfusion of autologous fresh plasma into peripheral vein, open approach |
|  | 30230M0 | Transfusion of autologous plasma cryoprecipitate into peripheral vein, open approach |
|  | 30230N0 | Transfusion of autologous red blood cells into peripheral vein, open approach |
|  | 30230P0 | Transfusion of autologous frozen red cells into peripheral vein, open approach |
|  | 30230R0 | Transfusion of autologous platelets into peripheral vein, open approach |
|  | 30230T0 | Transfusion of autologous fibrinogen into peripheral vein, open approach |
|  | 30230H1 | Transfusion of nonautologous whole blood into peripheral vein, open approach |
|  | 30230K1 | Transfusion of nonautologous frozen plasma into peripheral vein, open approach |
|  | 30230L1 | Transfusion of nonautologous fresh plasma into peripheral vein, open approach |
|  | 30230M1 | Transfusion of nonautologous plasma cryoprecipitate into peripheral vein, open approach |
|  | 30230N1 | Transfusion of nonautologous red blood cells into peripheral vein, open approach |
|  | 30230P1 | Transfusion of nonautologous frozen red cells into peripheral vein, open approach |
|  | 30230R1 | Transfusion of nonautologous platelets into peripheral vein, open approach |
|  | 30230T1 | Transfusion of nonautologous fibrinogen into peripheral vein, open approach |
|  | 30233H0 | Transfusion of autologous whole blood into peripheral vein, percutaneous approach |
|  | 30233K0 | Transfusion of autologous frozen plasma into peripheral vein, percutaneous approach |
|  | 30233L0 | Transfusion of autologous fresh plasma into peripheral vein, percutaneous approach |
|  | 30233M0 | Transfusion of autologous plasma cryoprecipitate into peripheral vein, percutaneous approach |
|  | 30233N0 | Transfusion of autologous red blood cells into peripheral vein, percutaneous approach |
|  | 30233P0 | Transfusion of autologous frozen red cells into peripheral vein, percutaneous approach |
|  | 30233R0 | Transfusion of autologous platelets into peripheral vein, percutaneous approach |
|  | 30233T0 | Transfusion of autologous fibrinogen into peripheral vein, percutaneous approach |
|  | 30233H1 | Transfusion of nonautologous whole blood into peripheral vein, percutaneous approach |
|  | 30233K1 | Transfusion of nonautologous frozen plasma into peripheral vein, percutaneous approach |
|  | 30233L1 | Transfusion of nonautologous fresh plasma into peripheral vein, percutaneous approach |
|  | 30233M1 | Transfusion of nonautologous plasma cryoprecipitate into peripheral vein, percutaneous approach |
|  | 30233N1 | Transfusion of nonautologous red blood cells into peripheral vein, percutaneous approach |
|  | 30233P1 | Transfusion of nonautologous frozen red cells into peripheral vein, percutaneous approach |
|  | 30233R1 | Transfusion of nonautologous platelets into peripheral vein, percutaneous approach |
|  | 30233T1 | Transfusion of nonautologous fibrinogen into peripheral vein, percutaneous approach |
|  | 30240H0 | Transfusion of autologous whole blood into central vein, open approach |
|  | 30240K0 | Transfusion of autologous frozen plasma into central vein, open approach |
|  | 30240L0 | Transfusion of autologous fresh plasma into central vein, open approach |
|  | 30240M0 | Transfusion of autologous plasma cryoprecipitate into central vein, open approach |
|  | 30240N0 | Transfusion of autologous red blood cells into central vein, open approach |
|  | 30240P0 | Transfusion of autologous frozen red cells into central vein, open approach |
|  | 30240R0 | Transfusion of autologous platelets into central vein, open approach |
|  | 30240T0 | Transfusion of autologous fibrinogen into central vein, open approach |
|  | 30240H1 | Transfusion of nonautologous whole blood into central vein, open approach |
|  | 30240K1 | Transfusion of nonautologous frozen plasma into central vein, open approach |
|  | 30240L1 | Transfusion of nonautologous fresh plasma into central vein, open approach |
|  | 30240M1 | Transfusion of nonautologous plasma cryoprecipitate into central vein, open approach |
|  | 30240N1 | Transfusion of nonautologous red blood cells into central vein, open approach |
|  | 30240P1 | Transfusion of nonautologous frozen red cells into central vein, open approach |
|  | 30240R1 | Transfusion of nonautologous platelets into central vein, open approach |
|  | 30240T1 | Transfusion of nonautologous fibrinogen into central vein, open approach |
|  | 30243H0 | Transfusion of autologous whole blood into central vein, percutaneous approach |
|  | 30243K0 | Transfusion of autologous frozen plasma into central vein, percutaneous approach |
|  | 30243L0 | Transfusion of autologous fresh plasma into central vein, percutaneous approach |
|  | 30243M0 | Transfusion of autologous plasma cryoprecipitate into central vein, percutaneous approach |
|  | 30243N0 | Transfusion of autologous red blood cells into central vein, percutaneous approach |
|  | 30243P0 | Transfusion of autologous frozen red cells into central vein, percutaneous approach |
|  | 30243R0 | Transfusion of autologous platelets into central vein, percutaneous approach |
|  | 30243T0 | Transfusion of autologous fibrinogen into central vein, percutaneous approach |
|  | 30243H1 | Transfusion of nonautologous whole blood into central vein, percutaneous approach |
|  | 30243K1 | Transfusion of nonautologous frozen plasma into central vein, percutaneous approach |
|  | 30243L1 | Transfusion of nonautologous fresh plasma into central vein, percutaneous approach |
|  | 30243M1 | Transfusion of nonautologous plasma cryoprecipitate into central vein, percutaneous approach |
|  | 30243N1 | Transfusion of nonautologous red blood cells into central vein, percutaneous approach |
|  | 30243P1 | Transfusion of nonautologous frozen red cells into central vein, percutaneous approach |
|  | 30243R1 | Transfusion of nonautologous platelets into central vein, percutaneous approach |
|  | 30243T1 | Transfusion of nonautologous fibrinogen into central vein, percutaneous approach |
| 68.39 | 0UT90ZZ | Resection of uterus, open approach |
| 68.49 | OUT97ZL | Resection of Uterus, Supracervical, Via Natural or Artificial Opening |
| 68.59 | 0UT97ZZ | Resection of uterus, via natural or artificial opening |
| 68.69 | 0UT90ZL | Resection of uterus, supracervical, open approach |
| 68.79 |  |  |
| 68.9x |  |  |
| 31.1 | 0B110F4 | Bypass trachea to cutaneous with tracheostomy device, open approach |
|  | 0B113F4 | Bypass trachea to cutaneous with tracheostomy device, percutaneous approach |
|  | 0B114F4 | Bypass trachea to cutaneous with tracheostomy device, percutaneous endoscopic approach |
| 96.70 | 5A1935Z | Respiratory ventilation, less than 24 consecutive hours |
| 96.71 | 5A1945Z | Respiratory ventilation, 24-96 consecutive hours |
| 96.72 | 5A1955Z | Respiratory ventilation, greater than 96 consecutive hours |
